# Glycosylated Triterpenoids as Endosomal Escape Enhancers in Targeted Tumor Therapies

**DOI:** 10.3390/biomedicines5020014

**Published:** 2017-03-29

**Authors:** Hendrik Fuchs, Nicole Niesler, Alexandra Trautner, Simko Sama, Gerold Jerz, Hossein Panjideh, Alexander Weng

**Affiliations:** 1 Institut für Laboratoriumsmedizin, Klinische Chemie und Pathobiochemie, Charité—Universitätsmedizin Berlin, Campus Virchow-Klinikum, 13353 Berlin, Germany; nicole.niesler@charite.de (N.N.); alexandra.trautner@charite.de (A.T.); hossein.panjideh@charite.de (H.P.); 2 Institut für Pharmazie, Freie Universität Berlin, 14195 Berlin, Germany; simkosama@zedat.fu-berlin.de (S.S.); weng@zedat.fu-berlin.de (A.W.); 3 Institut für Lebensmittelchemie, TU Braunschweig, 38106 Braunschweig, Germany; g.jerz@tu-braunschweig.de

**Keywords:** saponins, endosomal escape, efficacy enhancers, targeted toxins, immunotoxins, cytosolic drug delivery, controlled drug release, cancer treatment, endocytosis

## Abstract

Protein-based targeted toxins play an increasingly important role in targeted tumor therapies. In spite of their high intrinsic toxicity, their efficacy in animal models is low. A major reason for this is the limited entry of the toxin into the cytosol of the target cell, which is required to mediate the fatal effect. Target receptor bound and internalized toxins are mostly either recycled back to the cell surface or lysosomally degraded. This might explain why no antibody-targeted protein toxin has been approved for tumor therapeutic applications by the authorities to date although more than 500 targeted toxins have been developed within the last decades. To overcome the problem of insufficient endosomal escape, a number of strategies that make use of diverse chemicals, cell-penetrating or fusogenic peptides, and light-induced techniques were designed to weaken the membrane integrity of endosomes. This review focuses on glycosylated triterpenoids as endosomal escape enhancers and throws light on their structure, the mechanism of action, and on their efficacy in cell culture and animal models. Obstacles, challenges, opportunities, and future prospects are discussed.

## 1. Introduction

In the last decades new therapeutic strategies were developed in the battle against cancer including radio- and chemotherapeutic agents. Unfortunately these types of drugs exhibit strong side effects and operate in an unspecific manner, i.e., off-target cells are also affected. In consequence of that, the idea of a targeted tumor therapy was born, based on anticancer conjugates that obtain a target specific ligand function such as antibodies, growth factors or lectins, and a toxic moiety including radioisotopes, small molecule drugs or protein toxins [1]. Immunotoxins, which are targeted toxins that contain an antibody as targeting moiety, seemed to be very promising because they combine the specificity of an antibody against tumor-specific antigens, which enables them to channel the toxin to the point of action, and introduce additionally cell killing mechanisms such as antibody-dependent cell-mediated cytotoxicity and complement-dependent cytotoxicity [2,3,4]. To exhibit its effect, the toxin needs to be released into the cytosol after internalization. A major drawback is that the targeting moiety is often not fully internalized, directly recycled to the surface after internalization, or degraded in lysosomes [5,6,7,8]. To facilitate cytosolic entry of the toxin, it is possible to cleave off the toxin from the targeting moiety inside the cell by disulfide cleavage or endopeptidases [9,10]. Nonetheless, to insure a toxic concentration for tumor cells and to overcome insufficient cytosolic entry, high serum levels of the targeted toxin are required resulting in severe side effects, in particular including immunogenicity and vascular leak syndrome [11,12]. This demonstrates that new innovative strategies are urgently needed.

To cope with the drawback of insufficient cytosolic entry, many strategies were described including redirection of toxins to endogenous cellular membrane transport complexes of the biosynthetic pathway, disruption of endosomes, attenuation of the membrane integrity of endosomal membranes, or use of cell penetrating peptides [13,14,15,16]. Particular glycosylated triterpenes were found to act as tremendous endosomal escape enhancers for targeted toxins in tumor therapy [17,18,19]. These toxins essentially comprise ribosome-inactivating proteins (RIPs), a class of toxic plant enzymes that release a specific adenine residue from ribosomal RNA finally resulting in the arrest of protein synthesis and apoptosis [20]. Type 1 RIPs only consist of a single catalytically active polypeptide chain (A chain) while type 2 RIPs in addition possess a cell binding domain (B chain), making a number of these proteins highly cytotoxic, in particular ricin. Since the B chain recognizes regular cells, type 1 RIPs such as saporin, dianthin, agrostin, or gelonin are better suited for the design of targeted toxins. These toxins can be coupled to a targeting moiety by chemical conjugation or as a fusion protein [21]. The present review describes the different approaches to augment the efficacy of targeted toxins, especially of type 1 RIPs, by glycosylated triterpenoids. Structure–function relationships, investigations on the molecular mechanism of the endosomal escape, molecular interactions, cell culture experiments, and animal tumor models are presented. Pros and cons are discussed and the prospects and risks for future clinical development are outlined.

## 2. Glycosylated Triterpenoids

### 2.1. Origin and Structure

Saponins represent a wide spectrum in the field of secondary plant compounds and are subdivided into two groups, the steroid saponins and triterpenoid saponins. Occasionally, a third group of steroid alkaloid saponins is mentioned, however, often counted among the group of alkaloids [22]. Triterpenoid saponins are mostly found in eudicots [23,24], especially in families like Caryophyllaceae, Araliaceae, Fabaceae, and Hippocastanaceae [25].

Triterpenoid saponins are glycosides comprising a pentacyclic C30 terpene skeleton as a backbone and one or more covalently bound sugar chains [25]. The triterpene backbone of saponins is also called sapogenin or aglycone. Dependent on the number of sugar chains attached to the backbone, saponins are divided into mono-, bis-, and trisdesmosidic compounds. Generally, common sugars like glucose, fructose, galactose, and fucose as well as other sugars such as rhamnose, quinovose, and uronic acids are found in triterpenoid saponins. Linear as well as branched sugar chains are observed [22].

The wide range of structural variation options, both in the aglycone and sugar moieties explains the variety of different saponins with diverse effects. Nevertheless, all triterpenoid saponins can be described as amphiphilic due to the nonpolar triterpene backbone and the polar sugar chains. The ability of saponins to interact with both hydrophilic and lipophilic compounds enables them to interfere with or even disrupt cell membranes, phases and other amphiphilic structures [26]. These characteristics explain mostly all effects that are linked to saponins to date.

Saponins, the name derived from the Latin word “sapo”—soap, are soluble in water and create stable foam upon shaking [27]. Most saponins show hemolytic [28] and membrane permeabilizing [26] properties at specific concentrations, used by plants to defend themselves from predators. Below these toxic concentrations, triterpenoid saponins are widely used in several fields of phytotherapy. For instance, saponins derived from *Hedera helix* L. (ivy), *Glycyrrhiza glabra* L. (liquorice), and *Primula veris* L. (cowslip) are often used against productive cough [29] while triterpenoids from *Aesculus hippocastanum* L. (horse-chestnut) are known for their anti-inflammatory and anti-exudative effects [30,31], and *Panax ginseng* C. A. Mey. (Chinese ginseng) for blood flow stimulating effects [32].

In the recent past a new and considerable effect was observed for specific triterpenoid saponins. These saponins are able to substantially increase the cytotoxic effect of saporin, a type I RIP, in a synergistic manner [33]. Several studies showed that the cause for this effect is an increased uptake of the toxic compound into the cytosol when co-administered with triterpene saponins [34,35,36], however, this synergistic effect only applies for certain triterpenoids. Thus, many studies were conducted in order to demonstrate a structure–activity relationship in this respect.

In 2005, Melzig et al. [37] described the saponin-mediated enhancer effect for agrostin, another type I RIP, and also delineated the most important structural features of saponins required for this ability. An aldehyde function bound at C-4 and an acidic oligosaccharidic ester chain at C-28 play an important role in increasing the toxicity of agrostin. Monodesmosidic saponins showed lower toxicities in cell culture experiments, which underlined the importance of a C-28 glycosylation and the sugar chains in general. Bachran et al. [38] confirmed the previous findings of C-4 and C-28 as well as the vital role of the sugar side chains. Thus, highly active saponins usually are bisdesmosidic and possess a branched sugar chain at C-3 with a glucuronic moiety. Well-known representatives are oleanane saponins such as quillajasaponin from *Quillaja saponaria* Molina or Saponinum album from *Gypsophila paniculata* L., however, when combined with targeted toxins (i.e., toxins linked to a targeting moiety, e.g., an antibody), the specificity for target cells was lost when quillajasaponin was used [38] indicating further specific characteristics of particular saponins.

**Figure 1:**
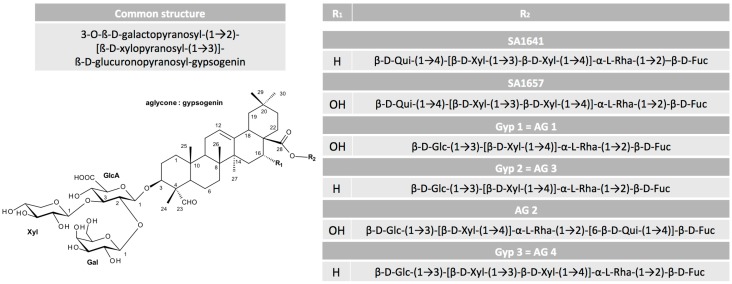
Relevant structural characteristics of the oleanane type triterpenoid saponins. The figure provides an overview on those saponins, for which the complete structure has been published to date. The residues at C-3 and C-4 and a sugar chain at C-28 with at least four sugar units are mandatory to enhance the cytotoxicity of protein toxins. The displayed differences in residues R_1_ and R_2_ determine the intensity of the effect. Fuc: fucose (6-deoxy-galactose); Gal: galactose; Glc: glucose; GlcA: glucuronic acid; Qui: quinovose (6-deoxy-glucose); Rha: rhamnose (6-deoxy-mannose); Xyl: xylose. Structure information was obtained for SA1641 from [40], for SA1657 from [41], for Gyp 1, Gyp 2, and Gyp 3 from [42], and for AG 1, AG 2, AG 3, and AG 4 from [43].

A valuable overview on the relation between structure and toxin enhancer ability of saponins was provided by Böttger et al. [39]. They corroborated previous findings and added more detailed information on the required sugar composition (Figure 1). The hydroxyl group at C-3 has to be linked to a branched trisaccharide comprising a glucuronic acid and the glycoside at C-28 must be a branched sugar chain. Finally, previous findings implied that for a drastic synergistic enhancer effect of saponins and protein toxins, a molecule mass of at least 1600 g/mol is necessary and aglycones consisting of an oleanane skeleton such as quillaic acid or gypsogenin are the most promising.

### 2.2. Purification

**Figure 2:**
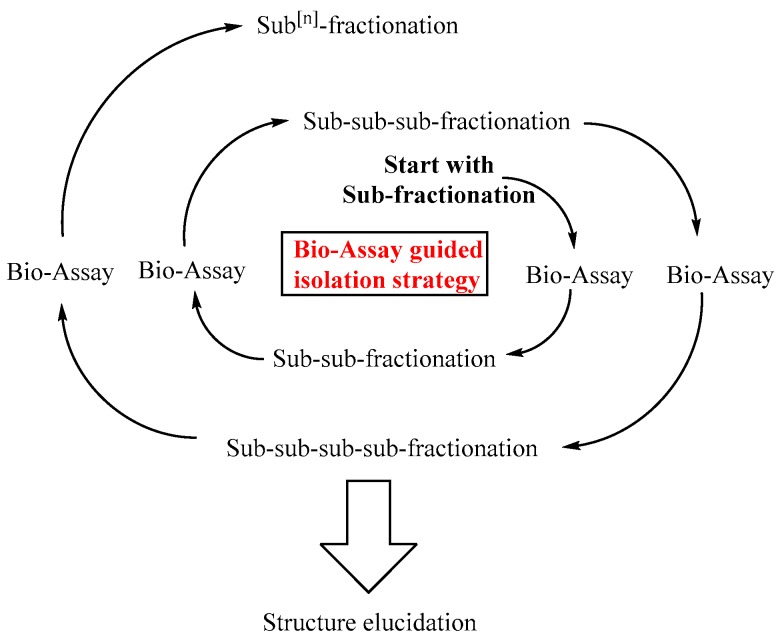
Bio-assay guided isolation of triterpene saponins. The bio-assay guided isolation allows the isolation and identification of biological active saponins. The raw extract is fractionated into sub-fractions, which are tested in suitable bio-assays. Bio-active sub-fractions are further purified resulting in a sub-sub-fractionation and so forth. Liquid chromatography–(tandem) mass spectrometry and high performance thin layer chromatography analyses are conducted at each point.

The isolation of chemically defined triterpene saponins is an extremely time-consuming task. The raw extracts used to isolate saponins are highly complex and comprise hundreds of different and partly very similar triterpene saponin structures. The main question that arises at this point is, which saponin should be isolated or to put it simply, which is the best saponin? From the medical point of view, the best saponins are those that enhance the endosomal escape of targeted anti-tumor toxins in tumor cells at a maximum while off-target cells remain unaffected. For this reason it is important to conduct series of corresponding bio-assays with targeted anti-tumor toxins and isolated triterpene saponins. The results of such bio-assays are important tools that guide the whole isolation process, leading to the identification of highly efficacious triterpene saponins. This concept is the basis of the bio-assay guided isolation strategy as exemplified in Figure 2.

The isolation of triterpene saponins starts with the preparation of the raw extract from the plant material such as roots or seeds. First of all, the roots are washed, freeze dried, and ground. Seeds are defatted by Soxhlet extraction with petroleum ether. Complex mixtures of mono- and bisdesmosidic triterpene saponins and carbohydrates (raw extract) are obtained by solvent extraction with water-methanol mixtures. The polarity of the solvent determines the composition of the raw extract. For instance an increased proportion of water in the solvent increases the proportion of carbohydrates in the raw extract. Generally, 90% methanol is used for the generation of the raw extract. The methanol is removed by vacuum distillation. The remaining saponin containing aqueous phase can be processed differently. Reducing the polarity of the solution, by adding acetonitrile or cold acetone, results in precipitation of the more polar triterpene saponins [44,45]. Alternative approaches comprise agarose gel-electrophoresis of the aqueous phase, resulting in pre-purified agarose-gel fractions that contain charged triterpene saponins [46,47,48]. An advantage of this technique is its simplicity. Dialysis of the aqueous phase is a method to reduce the complexity by excluding low molecular mass components such as carbohydrates [42].

After conducting one of the described methods, the pre-purified fractions are subjected to high performance liquid chromatography (HPLC). For this purpose, a C18 reversed phase column and methanol/water or acetonitrile/water gradients are used [49,50]. Trifluoroacetic acid is added at low percentages to repress the dissociation of uronic acids. In order to isolate single triterpene saponins several HPLC steps with different solvent compositions are combined. The isolation process is usually performed in the semi-preparative mode and each HPLC purification step is paralleled by a decrease of the yield. For each HPLC cycle, yields of around 1 mg can be achieved. Higher yields are only feasible by the time-consuming procedure of repeating HPLC cycles because at higher concentrations, beyond the critical micellar concentration, saponins form micelles. This phenomenon hinders the scaling-up of the isolation protocol for preparative amounts using preparative C18 HPLC columns. A solution for this problem is the application of countercurrent chromatography techniques (high-performance countercurrent chromatography, high-speed countercurrent chromatography, centrifugal partition chromatography), which are based on the separation of the triterpene saponins between non-miscible solvent layers [51].

**Figure 3:**
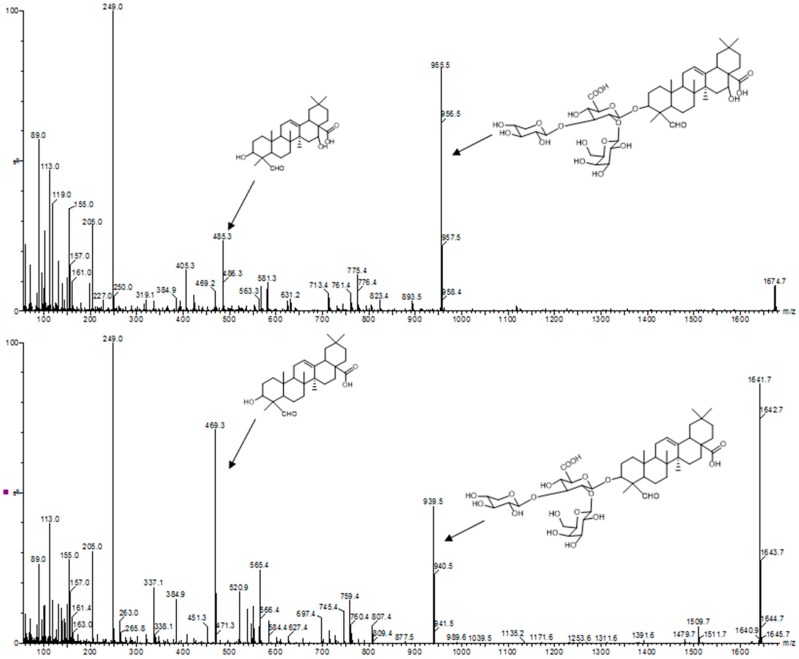
Tandem mass spectra of triterpene saponins. The triterpene saponin with *m*/*z* 1674 (SO1674) was isolated by countercurrent chromatography from the roots of *Saponaria officinalis* L. The triterpene saponin with *m*/*z* 1641 (SA1641) was isolated by HPLC from Saponinum album (Merck, Darmstadt, Germany). The identification of the aglycones of SO1674 (quillaic acid) and SA1641 (gypsogenin) was based on the fragmentation pattern. The peaks at *m*/*z* 955 and 939 were assigned to the prosapogenic parts of the bisdesmosidic saponins.

Triterpene saponins and saponin containing fractions are usually analyzed by high performance thin layer chromatography, thin layer chromatography-densitometry [52], infrared spectroscopy, and liquid chromatography coupled to (tandem) mass spectrometry. Tandem mass spectrometry is an indispensable tool that provides important structural information. By comparing the mass spectra of unknown saponins with the mass spectra of characterized triterpene saponins, it is possible to differentiate monodesmosidic from bisdesmosidic saponins. In addition, the corresponding aglycone can be identified (Figure 3).

### 2.3. Molecular Interactions

Saponins are initially considered to be part of the plant defense systems against pathogens and herbivores. Many other reports emphasize numerous biological functions [19] such as fungicidal [53], anti-microbial [54], insecticidal [55], and molluscicidal [56] activity and the enhancement of the endosomal escape and therefore their importance for targeted toxins [44,57].

#### 2.3.1. Hemolytic Activity of Saponins

Several studies revealed the ability of steroid and triterpenoid saponins as well as of many steroid alkaloid saponins to weaken membrane integrity [58]. It is assumed that this property is the molecular basis for the well-known ability of saponins to lyse mammalian erythrocytes and therefore often referred to as their hemolytic activity [59]. Dourmashkin et al. described this effect for the first time in their electron microscopic study on saponin-inactivated Rous sarcoma virus [60]. The essential role of cholesterol in the membrane of target cells for saponin-induced pore formation was subsequently reported by Bangham and Horne [61].

Augustin et al. perfectly reviewed the influence of the membrane composition on the function of steroid and triterpenoid saponins, and steroid alkaloid saponins [58]. In brief, the authors summarize that concentration and structure of incorporated membrane sterols affect the ability of glycosides to cause membrane perturbation. Moreover, all characteristics of the chemical composition of the glycosides themselves including structure of the aglycone, number and length of saccharide side chains as well as type and linkage variants of the incorporated sugar residues contribute to their hemolytic potential. However, the high number of structural characteristics that were found to modulate the hemolytic activity and the diversity in experimental setups resulted in partially conflicting conclusions [58].

#### 2.3.2. Interaction of Saponins with Cell Membranes and Their Components

In 1962 Bangham et al. proposed the first model of saponin actions towards membranes. They postulated that the interaction and formation of complexes between saponins and cholesterol in membranes lead to two-dimensional micellar-type structures within the membrane [61]. Other authors highlighted the hydrophilic origin of the saponins’ sugar chains that can form micellar-like complexes as aqueous pores. These pores allow higher permeability for ions and macromolecules. Augustin et al. described in their review the concept of pore formation and subsequent changes in ion conductivity and protein mobility through the membrane [58]. In brief, the first step of induced membrane-permeabilization is the incorporation of saponins into the membrane monolayer. The lipophilic character of the aglycone accelerates the interaction with hydrophobic membrane layers and the glycosides form complexes with membrane sterols, e.g., cholesterol. In addition, the interactions between the sugar residues of the incorporated glycosides with the membrane lead to a phase-separation. Subsequent accumulation of the glycosides within the cell membrane might cause membrane curvature and perturbation, and finally leads to pore formation and sterol extraction as underlined by electron microscopy [58]. In contrast, absence of pores was observed in cells treated with the steroidal glycoside digitonin [60]. Notably, pretreatment of cells with digitonin prevented formation of pores during subsequent treatment with pore-inducing saponins. Other investigations on digitonin revealed hemitubular alterations comparable to the finding of Keukens et al. for steroid alkaloid saponins [62,63]. Lin and Wang investigated the steroid saponin dioscin by computational simulations and demonstrated a curvature of the membrane that resemble hemitubular alterations [64] indicating the coexistence of pores and tubular alterations or vesiculation.

Since many studies demonstrated that saponins have different abilities to cause membrane perturbation and permeabilization, these phenomena were analyzed by Böttger and Melzig using a cell culture model and radioactive ^3^H-labeled cholesterol [65]. The authors revealed that cell membrane-active saponins (those with significant membrane toxicity) decreased the cholesterol content while no significant change in the cholesterol content was detectable after treatment with saponins that are not or only slightly cell membrane-active. Böttger and Melzig postulated a membrane toxicity mechanism that is caused by the saponin-dependent loss of membrane cholesterol. Notably, no significant effect of the investigated membrane-active saponins on the cholesterol of endosomes and lysosomes was measurable [65]. These results corroborated the observations of Krawczyk et al. who revealed the cell membrane-permeabilizing effect of digitonin, however, a saponin composite from *Quillaja saponaria* Molina in addition caused perturbation of intracellular membrane systems [66]. Lin and Wang described an alternative model of saponin activity towards membranes based on coarse-grained molecular dynamics simulations. Here dioscin interacts with sphingomyelin and cholesterol in the cell membrane and the subsequent enrichment of these complexes damages the structure of lipid rafts [64]. Lipid raft disruption and other microdomain alterations caused by saponins were also described by a number of other authors as reviewed by Augustin et al. [58].

In contrast, Segal et al. demonstrated that the presence of cholesterol is not essential for cell membrane permeabilization and pore formation mediated by certain saponins [67]. Real-time monitoring of cell membrane permeabilization was conducted to reveal the kinetics of saponin effects [68]. In this study, oleanane saponins showed variable permeabilizing effects on the cell and lysosomal membranes at distinct concentrations of 6 µM or higher (dependent on the saponin) and hemolysis at 3 µM or higher. The results further suggest that the charge of saponins might not play a significant role for their membrane permeabilizing effects. Böttger et al. described a correlation between the membrane toxicity of saponins and their ability to reduce the surface tension [26]. Apart from that, several structure–activity relation studies revealed that the power to cause hemolysis or membrane toxicity often does not correlate with the other known activities of saponins e.g., with their anti-fungal effects or their applicability as adjuvants [69,70].

An essential role of the acylation in the glycosidic part of saponins on metabolic stimulation was demonstrated by transient integration into the cellular membrane [71]. Another described mode of action for some saponins is their interaction with glucocorticoid receptors. Glucocorticoids are involved as steroidal hormones in the regulation of development, metabolism, neurobiology, and apoptosis pathways [72]. Therefore, several pharmacological activities such as anti-inflammatory and neuroprotective effects as well as induction of adipogenesis or apoptosis are linked to interactions of saponins with receptors of glucocorticoid hormones as reviewed by Augustin et al. [58].

Taken together, the biological activities of saponins are manifold reflected by their diverse chemical compositions and structures, and up to now the prediction of all their functions based on their composition is near to impossible [73].

## 3. Endosomal Escape in Cell Culture Models

### 3.1. Cellular Interactions

The most prominent general characteristics of saponins are their ability to form pores, to permeabilize the cell membrane [28,74] and to lower the surface tension of aqueous solutions [26,75]. Apart from these physico-chemical properties, saponins also possess pharmacological features [76], such as antimicrobial [54,77], immunomodulatory [46,78], anti-inflammatory [79,80], and anti-neoplastic [81,82] activities. A further promising field of application is their combination with anti-tumoral drugs. In many studies, additive or synergistic properties were described for saponins in combination with classical chemotherapeutic agents such as 5-fluorouracil [83] or cisplatin [84]. Other saponins showed no or only a slight increase in the effect of chemotherapeutic agents [85].

In 2003, Heisler et al. described for the first time that saponins are able to enhance the cytotoxicity of targeted toxins. The authors evaluated a combination therapy of a saponin composite (Saponinum album from *Gypsophila* species) and a targeted chimeric toxin composed of human epidermal growth factor (EGF) and saporin, a type I RIP [33]. Notably, the enhancer effect was observed at a non-permeabilizing concentration of the saponins. Another study by Hebestreit et al. underlined the saponin-mediated enhancer effect on the cytotoxicity of RIPs [35]. This phenomenon was diminished by latrunculin and bafilomycin, inhibitors of endocytosis and vacuolar-type H^+^-ATPase, respectively. On the contrary, the effect was neither altered by addition of various monosaccharides nor by brefeldin A that is known to dissolve the Golgi stacks. Saponins are able to only slightly enhance the toxicity of other peptidic toxins indicating a specific interaction with RIPs present in Caryophyllaceae [35]. Two years later Weng et al. found out that saponins from *Saponaria* spec. provoke clathrin-mediated endocytosis of saporin [86]. They used inhibitors of clathrin-mediated endocytosis, chlorpromazine, and imipramine, and significantly hampered the saponin-mediated cytotoxicity of saporin. Holmes et al. demonstrated that apoptosis is the mode of cell death in human leukemia and lymphoma cells induced by the combination therapy of triterpenoid saponins and immunotoxins [41]. The annexin V and propidium iodide data revealed again that the plasma membrane integrity of target cells was not affected by the saponins at the employed concentrations.

Kinetic studies by Weng et al. revealed that a small portion of saponin molecules remained associated with the cell after extensive washing [87]. These remaining saponins caused a drastic and long-lasting sensitization of the cells against saporin. This effect appeared to be dependent on the charge of the saponins as demonstrated by a charge-based fractional evaluation of saponins by Thakur et al. [48]. The authors revealed that only saponins of a certain electrophoretic mobility exhibited in agarose gels are able to enhance the endosomal escape of RIPs. Live cell imaging experiments applying such a saponin fraction with a relative electrophoretic mobility of 0.59 showed that these saponins mediate endosomal release of the toxic payload without affecting the membrane integrity and finally induce apoptosis while saponin fractions with other electrophoretic mobilities do not cause an effect. The enhancer effect of the active fraction was observed for type 1 RIPs but not for the investigated bacterial toxins. Therefore, Thakur et al. postulated electrochemical interactions between the saponins and the targeted toxins [48]. Moreover, Gilabert-Oriol et al. delineated a correlation between electrophoretic mobility and immune adjuvant, cytotoxic, and hemolytic properties of saponins [46]. The importance of the electrophoretic mobility for the saponin’s function comes along with the finding of a pH-dependent interaction between type 1 RIPs and saponins [36,40]. Using surface plasmon resonance, the authors observed that the investigated type 1 RIPs directly bind to saponins of the mentioned electrophoretic mobility at acidic pH while such binding did not occur at neutral pH. In the case of other toxins, such as ricin A chain and fragments of *Pseudomonas* exotoxin A and diphtheria toxin that were not enhanced by saponins, there was no binding at all. It can be hypothesized from this data that the direct interaction between saponins and type 1 RIPs at acidic pH is required for mediating the escape from late endosomes, particularly as neutralization of the endosomes results in loss of the enhancer effect [88], however, it must be noted that all the toxins that cannot be enhanced by saponins do naturally not reach the late endosomes so that intracellular trafficking might be more important than a direct interaction although the latter is conceivably a supporting property.

To characterize the enhancer effect of saponins in more detail, Thakur et al. purified two saponins with suitable electrophoretic mobility from *Gypsophila* species (SA1641, SA1657) and one from *Saponaria officinalis* L. (SO1861) [48]. SO1861 was more efficacious than SA1641 and SA1657, but all were active and the development of a reporter assay to measure the endosomal escape of toxin-based therapeutics by Gilabert-Oriol et al. underlined the postulated cytosolic release induced by SA1641 at a non-cytotoxic concentration [89]. Due to its high potential, SO1861 was further used in cell culture and in vivo studies (see following sections). Holmes et al. for the first time provided evidence that saponins are also able to enhance the effect of saporin-based immunotoxins in human leukemia and lymphoma cells [41]. They postulated a dual effect for the enhancer mechanism: first, a direct increase of the endosomal escape resulting in caspase-dependent apoptosis that is, second, combined with lysosomal-mediated cell death pathways, which are triggered after the release of cathepsins and other hydrolytic enzymes following destruction of lysosomal membranes by saponin/saporin complexes [41].

### 3.2. Endosomal Release of Proteins

**Figure 4:**
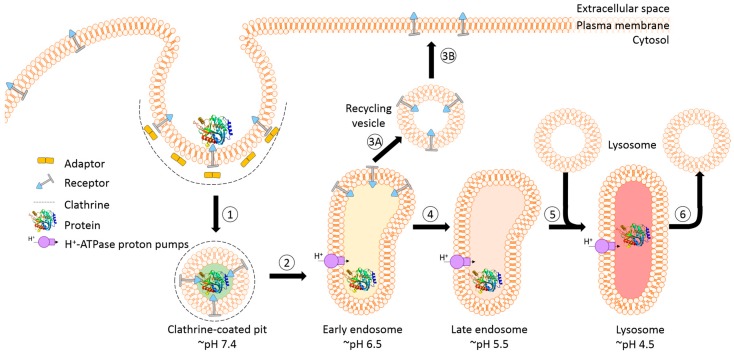
Schematic representation of receptor-mediated endocytic uptake of tumor-targeted proteins. (**1**) The first step is the binding of targeted toxins to their cognate receptor and internalization via the clathrin-dependent pathway; (**2**) The clathrin is removed and the uncoated vesicle fuses with the early endosome that mainly functions as a sorting compartment. Proton pumps cause a slightly acidic pH of 6.5 resulting (for most of the known receptor–ligand pairs) in the release of the ligand; (**3A**–**B**) The receptor and a large part of the membrane might be recycled back to the surface via recycling vesicles that bud from the endosome; (**4**) Proteins and substances destined for lysosomal degradation are transported by endosome carrier vesicles to late endosomes. The pH continues to drop to ~5.5; (**5**) The late endosome fuses with a lysosome, which results in the formation of an endolysosome and the pH further decreases to 4.5. Consequently, the protein gets degraded by proteases and cannot exhibit its function; (**6**) In a final step the lysosome is restored by budding off the hybrid organelle.

The success of protein-based targeted toxins for an efficient tumor therapy mainly depends on their ability to overcome the limited entry of the toxin into the cytosol of the targeted cell in sufficient quantity. Endosomal entrapment of molecules is a widespread problem not only for tumor-targeted therapy [57], but also in nanomedicine and gene therapy [90,91]. The internalized toxins are typically trapped inside endosomes and are either recycled back to the cell surface [92] or they become degraded in lysosomes [5,6,7,8] (Figure 4). The successful application of macromolecular drugs still remains a major bottleneck in targeted therapies since the internalized toxin needs to escape from the endosome in order to exhibit its function on the cytosolic target. Co-localization studies of a targeted toxin revealed that it preferentially accumulates in lysosomes of unaffected cells but not in sensitive cells [93]. This demonstrates that the process of lysosomal degradation is the most probable basis for resistance and emphasizes the need for the discovery of novel endosomal escape enhancers [13,57,93]. As a result of lysosomal degradation, relatively high concentrations of the targeted toxin are usually needed for modest cytosolic or nuclear delivery, leading to severe side-effects also for off-target cells [94]. Consequently, to increase the therapeutic window for tumor targeted toxins, the efficacy of delivery has to be enhanced and the following characteristics should be fulfilled: low immunogenicity and toxicity, minimized unspecific off-target effects, high efficacy and specificity as well as ease of use and production [13,57,95].

Many bacteria and viruses have evolved quite efficient strategies to successfully deliver their toxins or DNA into their targets. Understanding this mechanism of viral and bacterial escape from endosomes and exploring it for improving intracellular delivery has opened new avenues to overcome the endosomal barrier. These mechanisms include pore formation, membrane fusion, and the proton sponge effect [96,97,98,99]. Numerous developed protein delivery approaches mainly focus on selective disruption, and weakening or destabilization of the integrity of the endosomes [13,57]. This leads to an increased release of the macromolecular drug, for example by fusogenic peptides [100,101] or diverse chemicals including lysosomotropic amines [102,103], carboxylic ionophores [104,105] and calcium channel antagonists [99,106,107]. Another tool to mediate cytosolic delivery of proteins is the use of cell-penetrating peptides. These are short peptides often composed of cationic amino acids enabling the proteins to dive through the membrane [108,109]. A further strategy to overcome the endosomal entrapment of proteins can be achieved by the application of physiochemical processes, for example by light induced photochemical internalization [14]. In addition to all these approaches, another powerful and promising group of endosomal escape enhancers is the family of saponins as introduced in the preceding sections. Especially, saponins of the oleanane type possess the capability to enhance the endosomal release of several RIPs and RIP-based targeted toxins [19,34,37]. They synergistically enhance the cytotoxicity of different targeted toxins depending on the cell line by 3000 fold up to 4,000,000-fold in cell culture experiments [33,40,88,110,111]. The idea of using saponins in combination with a tumor targeted toxin containing a type 1 RIP arose from the finding that the combination of saponins and RIPs already exists in nature and might be evolved as a defense mechanism of the plants to protect themselves from herbivores [48,76].

The synergistic enhancement effect was first described for the type 1 RIP agrostin [34]. At that time, it was still hypothesized that the effect was probably based on the enhanced penetration of agrostin through the cell membrane when applied in combination with saponins. In the meantime several dozen saponins were investigated with regard to their ability to enhance the cytotoxicity of RIPs, but only a few of them with particular structural features were active [43]. These features include an aglycone of the oleanane type, a branched trisaccharide at C-3 containing a glucuronic acid, an aldehyde at C-4, a carboxy group at C-28, and a branched polysaccharide with at least four sugar residues also at C-28. One terminal sugar residue might be replaced by an acetyl group or 4-methoxycinnamic acid but none of the sugar residues must be modified with a bulky, long acyl chain [43]. In contrast to herein cited older studies, it is now accepted that the enhancer effect of saponins for RIPs neither occurs due to the permeabilization of the plasma membrane nor to the stimulation of endocytic events. Instead, saponins cause the escape of already internalized toxin molecules into the cytosol [112,113] and co-localization studies revealed that the relevant cellular compartments for the enhanced cytotoxicity are late endosomes and lysosomes [36,48]. This observation is of high importance for the use of such saponins in combination with targeted toxins (see next section) since target cell specificity is retained, which would not be the case if plasma membrane integrity is affected.

**Figure 5:**
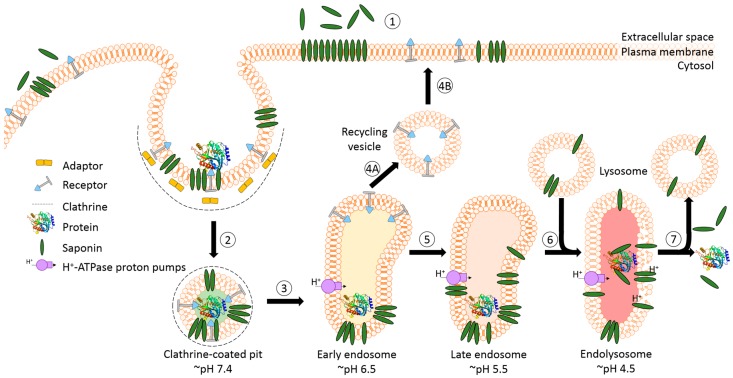
Model of the receptor-mediated endocytic uptake of a tumor-targeted protein in the presence of saponins with endosomal escape enhancer features. (**1**) The first step is the application of saponins prior to the targeted protein. The saponins integrate, due to their amphiphilic character, into the plasma membrane but do not affect the integrity, presumably because this particular group of saponins require an acidic pH; (**2**) The targeted protein binds to the cognate receptor and is internalized via the clathrin-dependent pathway. Saponins are taken up simultaneously together with the targeted protein and remain in the budded vesicle; (**3**) The clathrin is removed and the uncoated vesicle fuses with the early endosome; (**4A**–**B**). After acidic release of the protein, the receptor and a large part of the membrane might recycle back to the surface; (**5**) The targeted protein and membrane-associated saponins are transported via carrier vesicles to late endosomes; (**6**) The late endosomes fuse with lysosomes to endolysosomes. During the entire transport process, the pH continuously drops. The acidic pH in late endosomes and endolysosomes might initiate the association of saponins with the protein; (**7**) The saponins then mediate the endosomal release of the protein through the membrane into the cytoplasm. The molecular mechanism is not clear to date and appears to be a sophisticated interaction of cholesterol and other components of the membrane with the saponins and the protein triggered by low pH.

A prerequisite for saponin-mediated intracellular release of RIPs into the cytosol is an acidic pH. During the passage from early to late endosomes and endolysosomes, the pH gradually drops to an acidic milieu beginning at 7.4 (extracellular) and decreasing to ~6.5 (early endosomes), ~5.5 (late endosomes), and finally ~4.5 (endolysosomes) (Figure 5) [36,114]. It was assumed that saponins possessing the capability to mediate the endosomal escape are co-localized with the targeted toxins in the same vesicles [26]. It was hypothesized that inside these acidic vesicles, the saponins start to interact with the toxin component of the targeted toxin in a pH-dependent manner, modulating the endosomal release of the protein toxins through the membrane into the cytoplasm. The pH dependency was proven in a cell culture experiment by adding the inhibitors chloroquine or bafilomycin A1 to increase the pH prior to addition of a saponin composite [88]. This treatment resulted in a substantially reduced release of saporin into the cytosol of the targeted cell line. The effect was further confirmed for the purified saponins SA1657 and SO1861 [36]. Nevertheless, it is still unclear to date whether the acidic pH in late endosomes and endolysosomes initiates an association of saponin and RIP or only supports the disintegration of the membrane by saponins. The latter way is corroborated by the observations that saponins did not associate with the type 1 RIP gelonin at acidic pH although they can mediate an enhanced cytotoxicity of this toxin [115] and that saponins were also shown to have a great potential as a new transfection multiplier for non-viral gene delivery systems [45].

Although the approach to use saponins for an augmented endosomal escape appears to have unbeatable advantages, there are still some drawbacks that have to be solved. One major problem of saponins and most other chemical endosomal escape enhancers is that they are applied in all described experiments independently of the targeted toxin. They therefore behave differently with regard to body distribution, liberation, absorption, metabolization and excretion, and cellular internalization and release. This in turn can influence the efficacy of the combinatorial approach [57]. Cell culture experiments investigating the optimal order and time interval between application of saponins and targeted toxins delineated that the maximum synergistic toxicity is reached when the saponins are pre-incubated for five minutes and then followed by the administration of the targeted toxins [33]. These findings emphasize the importance of a synchronization of enhancer and protein kinetics to ensure that both compounds are at the same time at the site of interaction. Additionally it is worth noting that for in vitro studies, the non-lytic concentration for each cell line has to be determined individually [115]. Notwithstanding that there is a tremendous progress in the understanding of the enhancement of tumor targeted toxins by different saponins, more development and research is indispensable to completely comprehend and apply these secondary plant metabolites in combined therapies.

### 3.3. Synergistic Effects with Targeted Toxins

Targeted toxins are toxins that possess a binding domain for specific cell surface proteins. The binding domains of natural targeted toxins typically recognize normal cells and therefore exhibit counter-productive effects. Therefore, truncated versions that lack the natural cell binding domain were used to create tumor-targeted toxins. Examples are truncated versions of *Pseudomonas* *aeruginosa* (Schroeter) Migula exotoxin (PE40, PE38), diphtheria toxin (DT388, DT389, DT390) from *Corynebacterium diphtheriae* (Kruse) Lehmann and Neumann, and ricin (RTA) from *Ricinus communis* L. [116]. Truncated diphtheria toxin still possesses its native translocation domain to escape from early endosomes into the cytosol [117,118] while truncated *Pseudomonas* exotoxin employs a KDEL-related motive and thus follows retrograde transport to the endoplasmic reticulum to enter the cytosol [119]. Type 1 RIPs such as saporin (from *Saponaria officinalis* L.), dianthin (*Dianthus caryophyllus* L.) or agrostin (*Agrostemma githago* L.) consist, compared to type 2 RIPs such as ricin (*Ricinus communis* L.), abrin (*Abrus precatorius* L.) or nigrin (*Sambucus nigra* L.), only of an enzymatic active single A chain whereas type 2 RIPs additionally possess the cell-binding lectin B chain. This makes type 1 RIPs ideal candidates for tumor targeted toxins, however, due to a lacking mechanism for cytosolic entry, they are condemned to be degenerated inside endosomes and lysosomes [120,121]. As mentioned before, the effect of targeted toxins is dependent on the amount of toxin that has been released into the cytosol. To overcome these obstacles, enhancing agents are necessary. Several strategies exist to enhance the cytotoxicity of RIP-based targeted toxins such as photochemical internalization [122,123], insertion of a protein transduction domain [124], or usage of the pore-forming protein toxin listeriolysin O [125,126]. As described in the previous sections, another approach is the combinatorial application with glycosylated triterpenoids called saponins. When saponins gained the status of an enhancer for targeted toxins, most of the investigations were done with a saponin composite from *Gypsophila* species, Saponinum album. Due to the intricate structures contained in saponin composites, it was difficult to find out clear structure–function relationships. Different toxins were investigated in combination with Saponinum album, but only the cytotoxicity of agrostin and saporin was enhanced whereas no or nearly no effect was observed for diphtheria toxin, ricin toxin A chain, microcystin-LR, and nigrin [35]. It was assumed that the enhancement effect depends on certain conserved amino acids that are present in the RIPs dianthin, saporin, and gelonin but not in ricin A chain [115], nevertheless, the number of data is not sufficient for a final conclusion.

Of particular importance is the observation that the construction of a targeted toxin by fusion with EGF does not change the capability of a toxin to become enhanced by saponins. While EGF-PE40, DT390-EGF, and RTA-EGF only show enhancement factors of 1.2, 12.0, and 16.3, respectively, the cytotoxicity of EGF-saporin and EGF-dianthin was increased by more than four million times using saponin SA1641 [40]. This clearly indicates that the enhancement effect takes place inside the cell and thus, target cell specificity introduced by a ligand is retained. Considerable inhibitor studies showed that the increased cytotoxicity of saporin-EGF mediated by Saponinum album is clathrin- and actin-dependent. Six inhibitory agents that are known to inhibit either clathrin-mediated endocytosis, GTPase activity of dynamin-2, actin-polymerization, endosomal acidification, or caveolae-dependent endocytosis were tested [88]. Inhibition of clathrin-mediated endocytosis, actin-polymerization, and endosomal acidification blocked the enhancer effect of Saponinum album. The cytotoxicity of *Pseudomonas* exotoxin was again not affected. The investigations suggest that the retrograde pathway is not influenced by saponins, which would also explain why ricin toxin A chain is not enhanced. Diphtheria toxin is also not enhanced by saponins but in contrast to *Pseudomonas* exotoxin, diphtheria toxin enters the cytosol from early endosomes [127]. All these findings corroborate the model that the discussed subset of saponins acts in late endosomes and lysosomes. Solely the localization of the effect can explain why some toxins are enhanced and others are not. Direct interactions between saponins and toxins might play an additional role.

It is known that antibodies once they have been bound to the cellular receptor are internalized and then present in endosomes and lysosomes [128,129]. The cytotoxicity of a corresponding immunotoxin is hence dependent on the rate of endocytosis and the intracellular trafficking after internalization [130]. A colocalization study of Alexa Fluor-labeled saporin-trastuzumab in a live cell imaging experiment demonstrated that this immunotoxin is, in the absence of saponins, enriched in acidic vesicles such as endosomes and lysosomes [111]. The immunotoxins persist there, which finally leads to abrogation of their cytotoxic effect. Furthermore, a small amount of the protein was not internalized. However, after addition of the saponin SO1861 from *Saponaria officinalis* L. at a nontoxic concentration, the escape of saporin-trastuzumab out of the endosomes or lysosomes into the cytosol was induced. It is again important to mention that the cell membrane was not affected and the toxin remained inside the cell [111].

An important advantage of antibodies is their ability to trigger antibody-dependent cell-mediated cytotoxicity (ADCC) [131], which is initiated by the interaction of the Fc domain of the antibody with the FcγRIII of natural killer cells (NK) [132]. This important mechanism might be affected due to the chemical modification of the antibody with a toxin. Comprehensive impedance-based real-time viability assays with the antibodies trastuzumab and cetuximab as well as with their corresponding saporin-based immunotoxins were conducted with BT-474 (HER2-overexpressing) and HCT-116 (EGF receptor overexpressing) cells, respectively [115]. Both immunotoxins retained their ability to trigger ADCC with an efficacy comparable to that of the unconjugated antibody demonstrated in cell culture assays with freshly isolated NK-cells. The cytotoxicity of both conjugates was highly enhanced by adding a nontoxic concentration of SO1861, confirming the synergistic augmentation [115]. Furthermore, the binding affinity of the modified antibodies to their tumor-specific cellular antigens was not affected by toxin conjugation as displayed by surface plasmon resonance spectroscopy. The specificity of binding was confirmed by the inhibition of the cytotoxic effect in the presence of an excess of unconjugated antibody [111]. Similarly, a competitive fluorescence-based real-time binding assay of dianthin-cetuximab also exhibits high receptor specificity. After blocking of EGF receptors by unconjugated antibodies, the immunotoxin was not able to bind [133]. The application of saponins does not affect the binding specificity of these immunotoxins. Moreover, since the enhancement effect of saponins is not dependent on the targeting moiety as shown for a number of molecules including EGF, rituximab, cetuximab, trastuzumab, panitumumab, and obinutuzumab, the option to freely vary the ligands facilitates the development of a platform technology in the battle against solid and hematologic tumors.

While independent of the targeting moiety of the targeted toxin, the saponin-mediated enhancement factor of the cytotoxic effect is dependent on the cell line and on the expression and internalization level of the target molecule (Table 1) [33,38,40,41,44,110,133]. The enhancement factor is defined as the ratio of the concentrations in the absence and presence of saponins required for half maximal growth inhibition compared to untreated controls. The enhancement factor is different for varying targets (CD20, CD22, CD25) on the same cell line [44] and for the same target on a number of different cell lines [110]. The latter effect appears to be mostly dependent on target receptor expression, however, the enhancement factor decreased at very high amounts of the receptor. The reason for this is that at a certain surface concentration of the target receptor, the maximum possible cytotoxic effect has already been reached in the presence of saponins, i.e., further increase of the number of RIP molecules in the cytosol cannot inactivate more ribosomes (lack of substrate) [110]. Since growth inhibition further increases for higher target receptor expression in the absence of saponins (because only a low number of RIP molecules enters the cytosol so that an increase still has an effect), the enhancement factor begins to drop. As explained, this does not stand for quality loss. Since the endosomal escape effect of saponins depends on pH, the abnormal pH regulation in tumor cells [134] might also contribute to variations observed from cell line to cell line. When investigating non-specific enhancer effects of saponins by applying a targeted toxin on off-target cells and a ligand-free toxin on target cells, it was shown that there is some non-specific increase in cytotoxicity, however, this increase is much lower than the specific increase. The ratio of the enhancement factor for target cells and off-target cells was, dependent on the cell line, calculated to 56 (HER14), 342 (MDA-MB-435S), 367 (MCF-7), 760 (CaSki), 1514 (HeLa), and 50,000 (SiHa), indicating a specific augmentation of the cytotoxicity for target cells. Similar, the ratio of the enhancement factor for targeted cells treated by the targeted toxin and the toxin alone was 4.2–4.5 (HER14), 10 (CaSki), 19 (MDA-MB-435S), 30 (HeLa), 266 (MCF-7), and 500 (SiHa) [33,110]. Thus, the specificity for the enhancement is both ligand- and receptor-dependent. In addition, insertion of cleavable peptides, cell-penetrating peptides or ligand mutations into the targeted toxin did not influence the enhancer effect of saponins [135,136].

## 4. Endosomal Escape in Animal Models

### 4.1. Biodistribution and Toxicity

The convincing enhancer effect of saponins for targeted toxins in cell culture needs to be transferred to animal models for further development. In cell culture, both targeted toxins and enhancer are continuously present in the medium and therefore available for the cell at any time. But even in cell culture, the time interval between the application of the two substances and their order of application (saponin added before, simultaneously with or after targeted toxin) strongly influences the enhancer effect [33], indicating that uptake of saponins and targeted toxins into endosomes occurs independently via different routes and different kinetics. In animals, in addition other aspects such as distribution in the body, liberation, absorption, metabolization, and excretion influence the kinetics of the saponins and targeted toxins, raising the question of whether it is possible to synchronize their appearance inside the endosomes. Saponins with endosomal escape enhancer activity that are injected subcutaneously into the neck are rapidly distributed in the body with highest concentration in the kidneys after 10 min and in all other organs after 10 to 30 min. Nearly all of the saponin was found in the urine after 30 to 60 min and only about 1% was observed in the tumor between 10 to 240 min after injection [137]. It is assumed that part of the tumor-associated saponins become located inside the endosomes and are then able to mediate the endosomal escape. This process appears to take some time since injection of a targeted toxin results in a substantially lower effect when applied 10 min after saponins compared to 1 h [137].

In contrast to kinetic investigations, which require strong effort to detect and quantify the saponins in the different organs, toxicity studies are known for a number of saponins, however, most of the studies dealt with oral toxicity, which is not of interest for tumor treatment. Hemolysis, liver toxicity, and inflammation are the most prominent side effects of subcutaneously injected saponins. The hemolytic effect strongly depends on the structure of the saponins [46,68,138]. In vivo, the applied dose must be below the critical hemolytic concentration since otherwise progressive hemolysis will lead to death. Investigations on the toxic effects of an anti-leishmanial triterpene saponin showed that the target toxicity parameters leukocytosis, granulocytosis, lymphopenia, and the strong increase of the liver enzymes alkaline phosphatase, aspartate, and alanine aminotransferase did not return to normal pre-dose values within four weeks after subcutaneous dosing of 20 mg/kg for five consecutive days, but when injecting 5 mg/kg, the effects were marginal and doses of 2.5 mg/kg or lower did not differ from vehicle-treated control [139]. Subcutaneous injections of a saponin composite from *Gypsophila* species with endosomal escape enhancer properties showed toxic effects at 5 mg/kg and were lethal at 10 mg/kg, however, the typical dose of 0.75 to 1.5 mg/kg that is used for a combination therapy of saponins with targeted toxins is not toxic [140]. The saponin SA1641 purified from this *Gypsophila* saponin composite did not show any toxic effect at 5 mg/kg [36]. A more detailed analysis was done for SO1861 from *Saponaria officinalis* L. [141]. All animals treated with 10 mg/kg SO1861 died on the second day of the experiment. All animals treated with 5 mg/kg or less stayed alive for the entire observation period of 28 days. For some animals, a slight increase in aspartate and alanine aminotransferase activity, glutamate dehydrogenase activity and serum creatinine levels was detected in case of 3 mg/kg and 5 mg/kg, but no substantial increase in the average of these parameters was observed compared to placebo-treated controls [141]. Mild alterations of the liver (e.g., single cell necrosis) were detected by histopathological examinations in the majority of mice treated with 0.75–5 mg/kg SO1861. Less than 10% of these animals showed moderate liver damage (e.g., necrosis of small groups of cells). In contrast, severe injuries of the liver were often induced in mice receiving 10 mg/kg SO1861. In all cases, SO1861 did not damage kidneys and spleens, however, the spleens showed lymphatic hyperplasia indicating an immunogenic potential of the injected reagents [141].

### 4.2. Efficacy

The question was now whether endosomal enhancer saponins are able to improve the efficacy of an anti-tumor therapy with targeted toxins. Several tumor models were tested. Subcutaneous application of a particular saponin composite (1.5 mg/kg) and a saporin-based targeted toxin (0.005 mg/kg) in BALB/c mice bearing a solid tumor of mouse adenocarcinoma cells transfected with human epidermal growth factor receptor resulted in 94% tumor volume reduction and complete regression in seven of 10 cases [140]. Monotherapy with the same targeted toxin alone at the same concentration only resulted in 42% tumor regression and even with the 50-fold dose of 0.25 mg/kg only in 71% regression [124,140]. Side effects that find expression in observable complications, loss of body weight, altered blood parameters, histologic changes, and immune responses were only moderate and commonly reversible [140]. In a similar study with another saporin-based targeted toxin and the purified saponin SA1641, complete regression was observed in all cases [36]. When saponin SO1861 was used with the same targeted toxin, eight of 10 animals showed complete regression [141]. SO1861 was also tested in combination with a dianthin-based targeted toxin in a nude mouse model with subcutaneous tumors consisting of HCT116 human colon carcinoma cells [142]. Mice treated with 1.5 mg/kg SO1861 and 0.0175 mg/kg targeted toxin showed 96% tumor volume reduction and complete regression in three of four cases. Low-grade toxic alterations of the liver such as single cell necrosis and cytoplasmic degeneration were detected by histopathological analyses. Morphological alterations were neither observed in pancreas nor lungs. The lymphatic tissue of the spleen revealed a follicular hyperplasia in both verum and placebo group, which can be attributed to an immune response of residual T cells to tumor formation [142]. The same combination of saponin and targeted toxin was used for the treatment of pancreatic BxPC-3 cell carcinoma in nude mice [52]. There was an average reduction in the tumor volume of 97% and four out of five mice showed complete regression while monotherapy with the targeted toxin in absence of SO1861 only resulted in a 52% average reduction of the tumor and no complete regression (two mice had continuous tumor growth and three had retarded tumor growth). SO1861-induced skin hardening was observed at the injection site after two therapy cycles, but after six therapy cycles no skin lesions were observed either at the injection or at the tumor site. No altered values were detected in complete blood count analysis with the exception of increased platelet count [52]. Summing up all the mentioned studies, complete regression of the tumor was observed in 25 out of 32 mice treated with a saponin in combination with a targeted toxin. There are a number of other publications where saponins were used as enhancers in combination with other drugs in cancer therapy [18], however, in all these cases small molecule drugs were used, e.g., cisplatin, 5-fluorouracil, or paclitaxel, and the enhancer effect is based on other mechanisms, e.g., via cell cycle arrest, signaling pathways, and apoptosis [143,144].

## 5. Discussion

Surgery, conventional chemotherapy, and radiotherapy are the most relevant practices in the treatment of cancer. While surgery is limited to accessible tumors, chemotherapy and radiotherapy are often accompanied by severe side effects and formation of resistances. In the last decades more specific therapies mainly comprising receptor tyrosine kinase inhibitors [145] and antibody-based therapies [146] were developed. The efficacy of antibodies can be augmented by linking them to cytotoxic substances and other targeting ligands such as growth factors and cytokines can replace the antibody [147]. The toxic substances in protein-based drug systems are typically enzymes with an extremely high cell killing potential [148]. Type 1 RIPs are ideal toxins for targeted anti-cancer drugs since they are, once in the cytosol, highly toxic, and they can be coupled to any targeting moiety specific for tumor cells, however, these enzymes lack a mechanism to enter the cytosol. As described in this review article, a particular group of secondary plant metabolites that comprise saponins with certain structural elements were found to substantially enhance the endosomal escape of RIPs and to dramatically augment the efficacy of RIP-based targeted toxins in vitro and in vivo in mouse models [57]. The main question is now whether saponins are also a promising endosomal escape enhancer in the clinic.

Important requirements for saponins designated to be employed as endosomal escape enhancers can be formulated. (1) The natural source should be continuously available or alternatively, an economically reasonable method for the synthetic production of the substance should be known; (2) high grade purification and quality control must be possible; (3) long term stability must be guaranteed; (4) the saponins must be excretable or biodegradable; (5) they should not substantially interfere with metabolic processes of the organism; (6) they should not be toxic for regular cells; (7) they should not augment the uptake of the targeted toxin into off-target cells; and (8) their appearance at the site of action should be synchronized with the appearance of the targeted toxin.

The isolation of single pure products from complex natural mixtures is a challenging task, in particular because the composition of secondary metabolites can considerably vary in individuals of the same species dependent on growth conditions [149]. Therefore, high effort is required to ensure a defined and reproducible quality of the natural substance. There are two strategies to solve this problem, full chemical synthesis or production in plant cell bioreactors. Chemical synthesis is possible, however, the sophisticated structure of saponin-based endosomal escape enhancers requires comprehensive multistep syntheses, which are presently not economical [150]. Nevertheless, an indisputable advantage of the chemical synthesis is the option to modify and optimize the structure with regard to efficacy, side effects, immunogenic potential, size, and intricacy. An alternative to full synthetic products is the synthesis of saponin analogues from building blocks obtained from natural sources [151]. Bioreactor production makes the process independent of natural sources and allows control of preferred products by additives, however, requires high investment [152,153].

The biological properties of saponins as endosomal escape enhancers mostly meet the needs. They are stable, excretable, biodegradable, and do not interfere with important metabolic pathways. As also reported in this review, they are not toxic when applied at concentrations not higher than required for the augmentation of the endosomal escape. Nevertheless, a common feature of all these substances is that they are per se not target-specific and distribute with other kinetics than the targeted toxins. Thus, after application, the saponins can be found in any organ [137] connoting that specificity is only mediated by the targeted toxin. If the toxin affects off-target cells, it is also augmented in these cells. Moreover, distribution of saponins in the whole body requires higher concentrations for a successful treatment as in the case of specific accumulation in target cells. The latter would improve the safety profile of saponins but would not be able to solve the problem of different pharmacokinetics. A lot of effort is necessary to find practicable solutions.

The mechanism of saponin-mediated endosomal escape is not fully understood. As described before, a lot of descriptive data is available that helps us to understand structure–function relationships, however, at the moment we are not in the situation to predict what we have to change in the saponins, in the targeted toxins, and in the treatment regimen to further improve the efficacy and tolerance. For instance, it was observed for a number of RIPs that they directly interact with saponins at acidic pH and it was concluded that this might explain the enhancer effect, but in the meantime, other molecules were found that are enhanced by saponins [45]. It has not yet been proven, but all the data together appears to imply that the intracellular trafficking into endosomes or endolysosomes determine whether cytosolic entry of a substance can be enhanced by saponins. Further understanding of the mechanism of how these particular saponins manage to make the endosomal membrane somewhat leaky without affecting other membranes will enable us to develop a more rational design of this drug delivery system.

## 6. Conclusions

Particular saponins with certain defined structural features are potent molecules in order to enhance the endosomal escape of tumor cell targeted toxins. This substantially improves the efficacy of the toxins in vitro and in mouse tumor models. The saponins do not impair the integrity of the plasma membrane so that the specificity of the targeted toxins for cancer cells is retained although the saponins are distributed in the whole body and also affect off-target cells. Compared to the monotherapy with targeted toxins, the combination therapy has less side effects since the toxin can be applied at very low concentrations and the saponins are employed at non-toxic levels. The major problem is the synchronization of the pharmacokinetics of the two substances to deliver them at the same time to the endosomes of the target cells. Solving this challenge might result in a promising new general drug delivery system for cytosolically active macromolecules.

## Figures and Tables

**Table 1:** Enhancement factors for saponin-mediated cytotoxicity on different cell lines. GI50 refers to half maximal growth inhibition compared to untreated cells.

Toxin	Ligand	Saponin	GI50 withoutSaponin [nM]	GI50 with Saponin [nM]	Enhancement Factor	Cell Line	Reference
Dianthin	EGF	SA1641	0.45	<0.0000001	>4,000,000	HER14	[40]
	Cetuximab	SO1861	>10	0.0053	>1886	HCT116	[133]
	Panitumumab		>10	0.0015	>6666	HCT116	
	Trastuzumab		>10	0.023	>434	BT-474	
Saporin	EGF	Saponinum album	80	0.0011	76,000	PHCC1	[110]
			24.5	0.0027	9000	PHCC2	
			>300	0.00012	>2,500,000	SiHa	
			53	0.0007	75,700	HeLa	
			5	0.00013	38,000	CaSki	
			2.5	0.0009	2800	HER14	
			206	0.012	17,100	MDA-MB-435S	
	Adapter-EGF	Quillaja saponin	2.4	0.0017	1434	HER14	[38]
			27.2	0.013	2113	NIH-3T3	
		Saponinum album	2.4	0.00018 0.00067	13,647 3560	HER14	[33,38]
			27.2 135	0.014 0.13	1977 1050	NIH-3T3	
			1040	0.0027	385,000	MCF-7	[33]
	EGF	SA1641	57	<0.0000001	>4,000,000	HER14	[40]
	Rituximab	SO1861	7	0.01	700	Ramos	[44]
	Anti-CD22		0.5	0.003	170		
	Anti-CD25		1	0.04	25		
	HB2	Saponinum album	139	0.31	448	Daudi	[41]
			1000 *	0.88 *	1130	Ramos	
			0.5 *	0.003 *	146	HSB-2	
	BU12		965	0.00003	31,500,000	Daudi	
			1.5 *	0.000867 *	1730	Ramos	
			250 *	0.08 *	3140	HSB-2	
	4KB128		0.0139	0.0000226	615	Daudi	
			0.15 *	0.0000331 *	4520	Ramos	
			1000 *	25 *	39	HSB-2	
	OKT10		0.0532	0.000222	242	Daudi	
			1 *	0.000346 *	2890	Ramos	
			0.2 *	0.0007246 *	276	HSB-2	
	DF1513		0.0143	0.0000413	346	Daudi	
			0.8 *	0.000396 *	2020	Ramos	
			0.03 *	0.0001724 *	174	HSB-2	

* Estimated number obtained from Figure 5 of the corresponding reference. EGF: epidermal growth factor; HER14: embryonic mouse fibroblasts (NIH-3T3) stably transfected with human epidermal growth factor receptor; HCT116: human colorectal carcinoma cells; BT-474: human breast ductal carcinoma cells; PHCC: human cervical carcinoma cells; SiHa: human cervical squamous carcinoma cells; HeLa: human cervical adenocarcinoma cells; CaSki: human cervical epidermoid carcinoma cells; MDA-MB-435S: human breast carcinoma cells; NIH-3T3: embryonic mouse fibroblasts; MCF-7: human breast adenocarcinoma cells; Ramos: human Burkitt's lymphoma cells; Daudi: human Burkitt's lymphoma cells; HSB-2: human lymphoblastic leukemia cells.
